# Dr. Fisher’s Case

**Published:** 1860-05

**Authors:** 


### DR. FISHER'S CASE.

Dr. Fisher has brought a suit against H. O. Stone, of this city, for libel. The ground alleged is, that Dr. F. attended Mrs. Stone in labor; some time afterward she was found to have invertion of the womb—Stone accused Fisher of some mismanagement; hence the suit. Dr. Fisher published in this Journal, his version of the matter; Dr. Potter, of Geneva, N. Y., some time since, published another, probably Stone's version.

There the matter stood, when we noticed in the last number of the *Cleveland Medical Gazette*, a report of the deposition of Prof. John Delameter, taken, it is presumed, to be used in the case. We know not whether this deposition is intended or expected to favor the plaintiff or the defendant in this case. Its publication at this time, is calculated, if not intended, to manufacture public opinion and thus prejudge the case. If the publication was made in this state, it might be easily stopped by injunction—out of the state, it cannot. We respectfully suggest to the highminded proprietors of the "Gazette," whom we truly respect, whether the cause of justice is likely to be better served by the publication of *ex-parte* testimony, than by laying it all before their readers after it has been used in court.

What would the editor say if a Chicago publication should take a course calculated to influence, in any way, the action of a court in Cleveland?

We claim for the courts, the bar, and the medical profession of Chicago, as much integrity, learning, and capacity as are possessed by those of any other city of its size. We think they can administer justice or aid in its administration, without dictation from any quarter.

